# Graphene Quantum Dots Decorated Al-Doped ZnS for Improved Photoelectric Performance

**DOI:** 10.3390/ma11081452

**Published:** 2018-08-16

**Authors:** Zheng Zhang, Yun Lei, Liyang Zhao, Zicong Jiang, Zhong Ouyang

**Affiliations:** 1 School of Resources and Environmental Engineering, Wuhan University of Technology, Wuhan 430070, China; 219915@whut.edu.cn (Z.Z.); whut_jzc@163.com (Z.J.); oyzhong1995@163.com (Z.O.); 2 Hubei Entry & Exit Inspection and Quarantine Bureau Technology Center, Wuhan 430050, China; whqsghft@163.com

**Keywords:** Al doping, Al-ZnS/GQDs, photocurrent-time test, electrochemical impedance spectra

## Abstract

Graphene quantum dots (GQDs) decorated Al-doped ZnS composites were prepared using the solvothermal process, and the hydrothermal method was used to prepare GQDs. Various spectroscopic techniques were used to characterize the products, and the results show that Al-ZnS attached GQD composites present lattice fringes that can be assigned to ZnS and GQDs, respectively. The absorption peaks of Al-ZnS/GQDs are red-shifted because of the doping of aluminum and the incorporation of GQDs. The luminescence intensity of Al-ZnS/GQDs shows a downward trend with the addition of GQDs. As the GQD content changes from 0.6 wt % to 1.8 wt %, the photocurrent density achieves a maximum at the addition of 1.2 wt %. The photocurrent of Al-ZnS/GQDs composites are about 700% and 200% of pure ZnS and Al-ZnS, respectively. The results indicate that Al doping can reduce the energy bandgap of ZnS and produce more photogenerated electrons. The photogenerated electrons from Al-ZnS can be extracted and transferred to GQDs, which act as conducting materials to decrease the recombination rate and improve the photogenerated electron-transfer.

## 1. Introduction

ZnS is one of the promising direct-band semiconductors and is extensively investigated due to its wide bandgap, large exciting binding energy, and superior photoelectric characteristics [1,2,3,4]. When the light was irradiated on the surface of ZnS, the photogenerated electrons were limited by a broad bandgap of ZnS semiconductor. In order to solve the problem, reducing the bandgap by doping other elements is required [5,6,7,8]. Jie et al. developed a thermal co-evaporation route to synthesize Al-doped ZnS nanowires with large gain-bandwidth and high responsivity [9]. Reddy et al. prepared the ZnS:Al nanostructures, and investigated the variation of the morphological, structural, optical, and photoluminescence properties caused by Al doping. [10]. Dole et al. used a hydrothermal method to synthesize Al doped ZnS nanorods, and the energy bandgap of Al doped ZnS shows a red shift [11]. Bacha et al. prepared Al doped ZnS thin films using a spray paralyzes method. The optical energy gap of Al doped ZnS decreases with doping concentration [12]. The reports indicate that Al doping narrows the bandgap and facilitates the generation of electron-hole pairs. However, the photogenerated electrons are easily recombined, which limits the photoelectric properties of Al-doped ZnS. 

GQDs, as a new kind of carbon materials, have been increasingly attractive due to their low cost, small size, edge effects, and high conductivity [13,14,15]. GQDs are considered as excellent electron conductors to combine with semiconductor materials because GQDs can effectively promote the rapid transfer of electrons and prevent the recombination between electrons and holes [16,17,18]. Pan et al. reported a hydrothermal deposition method to fabricate GQDs-TiO_2_ heterojunctions with higher cycling stability and visible-light photocatalytic activity [19]. Narula et al. employed a chemical polymerization method to synthesize poly(3,4-ethylenedioxythiophene)@ZnO@GQDs composites with excellent photocatalytic properties [20]. Min et al. prepared CdS/GQDs nanohybrids by a hydrothermal method, and the nanohybirds can be used as a kind of photocatalyst driven by visible-light [21]. Shi et al. combined CdSe with GQDs to magnify the electrochemiluminescence intensity of CdSe and reduce the onset potential of electrochemiluminescence reaction [22]. Yu et al. added a small amount of GQDs during the process of spin-coating the SnCl_2_ and attained SnO_2_:GQD electron-transporting layers. The electron trap can be filled and the electron density can be improved through the electron transfer from the GQDs to SnO_2_ [23]. In our previous work, ZnS/GQDs were synthesized via a solvothermal process, and the products presented photocurrent enhancement [24]. A continuing effort was proposed to combine Al-doped ZnS with GQDs to reduce the energy bandgap and prevent the recombination between the photogenerated electrons and holes, thereby improving the photoelectric performances of Al-doped ZnS.

In this work, the hydrothermal method was employed to fabricate the GQDs, and one-step solvothermal synthesis was used to prepare the Al-ZnS/GQDs composites. The products were characterized by X-ray diffraction (XRD), X-ray photoelectron spectroscopy (XPS), transmission electron microscopy (TEM), ultraviolet-visible absorption spectroscopy (UV-vis), photoluminescence spectra (PL), and Fourier transform infrared spectra (FTIR). Photoelectric characteristics were investigated via photocurrent-time test and electrochemical impedance spectroscopy (EIS).

## 2. Materials and Methods 

### 2.1. Materials

Graphite powders (99%), sodium nitrate (NaNO_3_), potassium permanganate (KMnO_4_), hydrogen peroxide (H_2_O_2_), sulfuric acid (H_2_SO_4_), anhydrous ethanol (CH_3_CH_2_OH), nitric acid (HNO_3_), sodium hydroxide (NaOH), ethylene glycol (CH_2_OHCH_2_OH), thiourea (SC(NH_2_)_2_), aluminum nitrate (Al(NO_3_)_3_·9H_2_O), zinc acetate (Zn(CH_3_COOH)_2_·2H_2_O), and polyethylene glycol (PEG) were of analytical grade.

### 2.2. Fabrication of GQDs 

Hydrothermal method was used to synthesize the GQDs. At first, graphite oxide was prepared by a three-stage Hummers method and thermally reduced to graphene sheets which were added into a mixture (volume ratio, sulfuric acid: nitric acid= 1:3)and sonicated for 10 h. The mixture was diluted and centrifuged with distilled water to remove the acid. Then the dry sample was dissolved in distilled water through ultrasonication to obtain a homogeneous mixture. NaOH solution (0.1 M) was dropped into the homogeneous mixture to adjust the pH value of 8. The prepared mixture was autoclaved at 200 °C for 10 h. At last, the mixture containing GQDs was filtered through microporous membranes (0.22 μm) to obtain pale yellow filtrate, and this filtrate was dialyzed (retained molecular weight: 3500 Da) for 24 h to get GQDs.

### 2.3. Synthesis of ZnS, Al-ZnS, Al-ZnS/GQDs Composites 

Solvothermal process was used to synthesize the Al-ZnS/GQDs composites. Zinc acetate and thiourea were used as Zn and S precursors, respectively. Aluminum nitrate was used as Al dopants. At first, zinc acetate, thiourea, aluminum nitrate, and GQDs were dispersed in ethylene glycol. Subsequently, the mixture was placed into an autoclave and kept the temperature at 175 °C for 8 h. At last, the mixture was centrifuged with distilled water and followed drying at 45 °C to obtain Al-ZnS/GQDs. 

Al-ZnS/GQDs (Al-ZG) composites with different GQDs content were prepared by changing the content from 0.6 wt % to 1.8 wt %. The composites were denoted as Al-ZGX, where ‘X’ was the GQDs content of 0.6 wt %, 1.2 wt % and 1.8 wt %. Pure ZnS (no Al dopants and GQDs during preparation) and Al-ZnS (no GQDs during preparation) were prepared in the same solvothermal process.

### 2.4. Characterization 

XRD was performed on a D8 Advance diffractometer (Bruker, New York, NY, USA) using CuKα1 radiation with λ = 1.5406 Å. XPS was obtained at an ESCALAB 250XI electron spectrometer (ThermoFisher, Waltham, MA, USA). The morphology of the samples was investigated by a JEM-2100F transmission electron microscope (JEOL, Tokyo, Japan). UV-vis spectra were analyzed on a UV5500 spectrophotometer (Shimadzu, Kyoto, Japan). PL spectra were investageted on a Cary Eclipse fluorescence spectrophotometer (Varian, Palo Alto, CA, USA). FTIR spectra were collected with an IS-10 FTIR spectrometer (Nicolet, Madison, WI, USA). Photocurrent-time test and EIS were investigated in a three-electrode system via a CHI650E electrochemical work station (Chenhua, Shanghai, China).

### 2.5. Photocurrent Measurements

Photocurrent measurements were investigated in a three-electrode system. The platinum foil was worked as the counter electrode and the saturated calomel electrode was worked as the reference electrode. The working electrode was composed of FTO (F-doped SnO_2_) glass coated with the sample. At first, sample and PEG (mass ratio, sample: PEG = 1:2) were dispersed in small amount of ethanol via grinding to form a homogeneous mixture. Then, the mixture was evenly coated on the FTO glass and annealed at 350 °C in N_2_ environment for 30 min. Transient photocurrent responses measurements were conducted at an on/off cycles of intermittent light irradiation (60 s).

## 3. Results and Discussion

### 3.1. XRD Analysis

**Figure 1:**
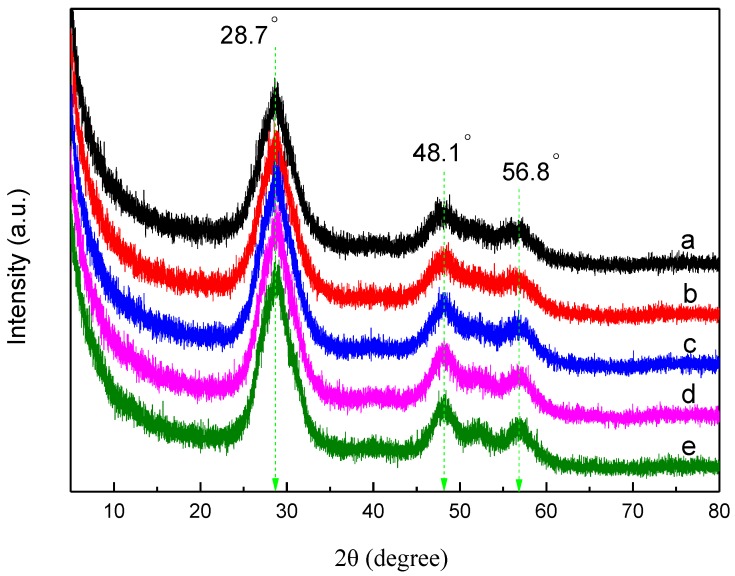
XRD patterns of pure ZnS (**a**), Al-ZnS (**b**), Al-ZG0.6 (**c**), Al-ZG1.2 (**d**) and Al-ZG1.8 (**e**).

Figure 1 represents the XRD patterns of pure ZnS, Al-ZnS, and Al-ZG composites. There are three obvious diffraction peaks at 28.7°, 48.1°, and 56.8°, which reveal the coincident diffraction peaks indexed to cubic ZnS (JCPDS no. 77-2100). The diffraction peaks of Al-ZnS are similar to those of pure ZnS, and the result proves that the incorporation of a small amount of Al has no effect on the structure of ZnS. Furthermore, the diffraction peaks of Al-ZG are also similar to those of pure ZnS, which suggests that the broad peak at 25° is too weak to be covered by the strong peak at 28.83° indexed to the (111) plane of ZnS. 

### 3.2. XPS Analysis

**Figure 2:**
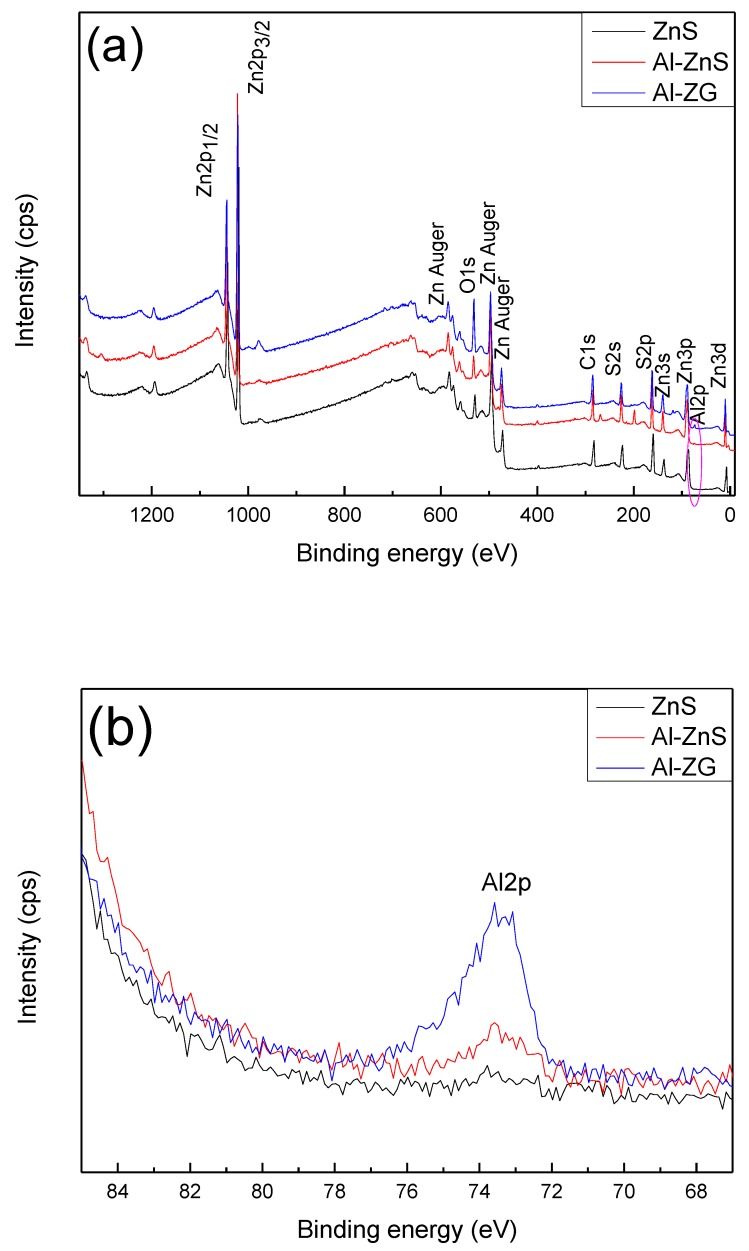
(**a**) Survey and (**b**) Al 2p spectra of pure ZnS, Al-ZnS and Al-ZG.

The surface chemical compositions of ZnS, Al-ZnS, and Al-ZG composites were measured by XPS and the results were shown in Figure 2 and Table 1. As shown in Figure 2a, the survey XPS spectrum of Al-ZG indicates the presence of zinc, sulfur, aluminum, carbon, and oxygen. The peaks at 1042 and 1019 eV in Figure 2a are assigned to the binding energies of Zn 2p_1/2_ and Zn 2p_3/2_ of pure ZnS, respectively. As shown in Figure 2b, the peak at 73.58 eV belongs to the binding energy of Al2p on the surface of Al-ZnS and Al-ZG composites. The intensity of the peak energy among Al-ZnS and Al-ZG is obviously enhanced, which suggests the successful doping of Al into ZnS. 

### 3.3. TEM Analysis

**Figure 3:**
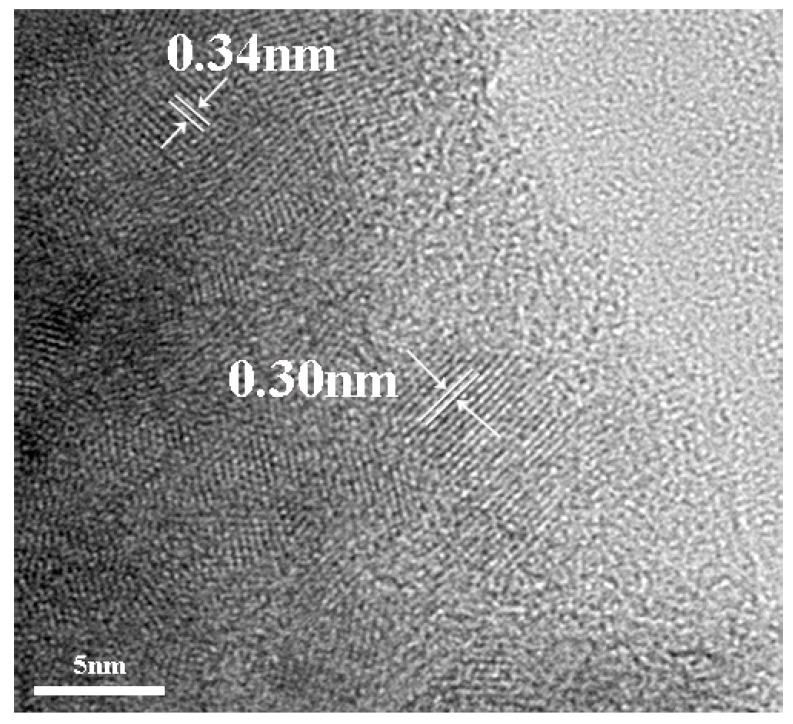
TEM image of Al-ZG.

TEM analysis was used to characterize the morphology of Al-ZG composites. As shown in Figure 3, GQDs and Al-ZnS are approximately 5–10 nm in size. Al-ZnS attached GQD composite present lattice fringes that can be assigned to ZnS and GQDs. The lattice spacing of 0.30 nm belongs to the (111) crystal facets of ZnS, and the lattice spacing of 0.34 nm belongs to the (002) crystal facets of GQDs [16,25].

### 3.4. UV-vis Absorption Spectra

**Figure 4:**
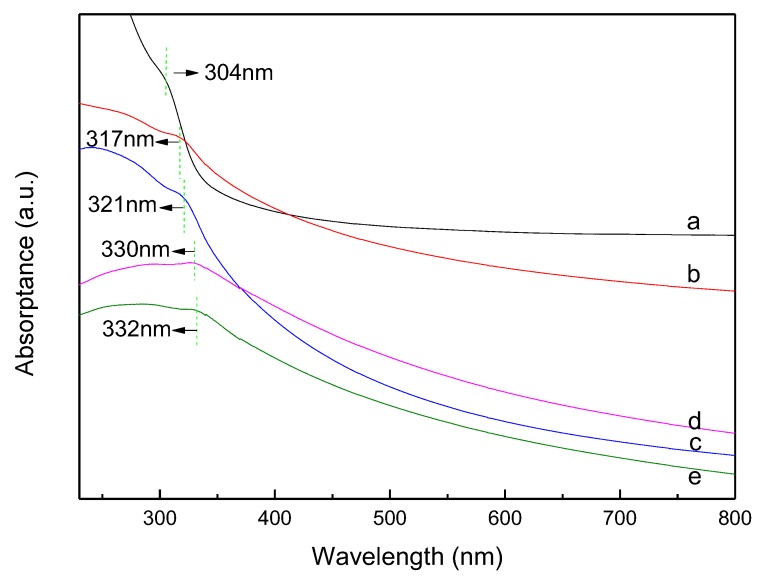
UV-Vis absorption spectra of ZnS (**a**), Al-ZnS (**b**), Al-ZG0.6 (**c**), Al-ZG1.2 (**d**) and Al-ZG1.8 (**e**).

The absorption spectra of ZnS, Al-ZnS, Al-ZG0.6, Al-ZG1.2, and Al-ZG1.8 are shown in Figure 4. An evident shoulder appeared at 304 nm, which belongs to the characteristic absorption of ZnS. The absorption peak of Al-ZnS shifts to 317 nm as compared with the ZnS, which can be assigned to the exchange interaction between the d electrons of the aluminum ions and the s and p electrons of the host electron band. [11]. With the addition of GQDs, the absorption peaks of Al-ZG0.6, Al-ZG1.2, and Al-ZG1.8 are further red-shifted to 321 nm, 330 nm, and 332 nm, respectively. This red-shift might be ascribed to the interaction between GQDs and Al-ZnS [26].

### 3.5. PL Spectra

**Figure 5:**
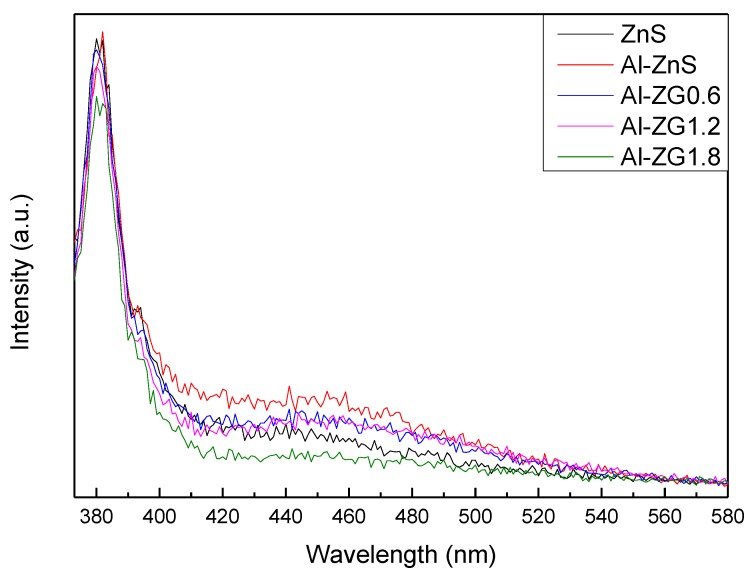
PL spectra of ZnS, Al-ZnS, Al-ZG0.6, Al-ZG1.2 and Al-ZG1.8.

The PL spectra of ZnS, Al-ZnS, Al-ZG0.6, Al-ZG1.2, and Al-ZG1.8 were investigated and the results are shown in Figure 5. Pure ZnS has an emission peak centered at about 381 nm, a shoulder at 394 nm, and a broad emission peak at about 446 nm. These emission peaks can be attributed to zinc interstitials, surface sulfur vacancies, and the intrinsic emission of defects, respectively. An enhanced luminescence can be observed in Al doped ZnS compared to the pure ZnS due to the increase of electrons in the conduction band and the decrease of the band gap. With the addition of GQDs, the luminescence intensity shows a downward trend due to the reason that the GQDs can act as an excellent electron conductor to transfer the electrons to the external circuit and prevent the recombination of electron-hole pairs.

### 3.6. FTIR Transmission Spectra

**Figure 6:**
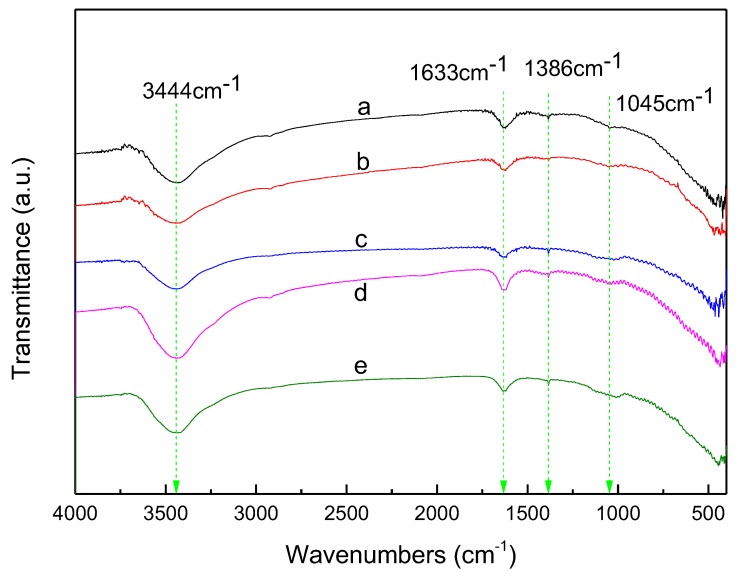
FTIR transmission spectra of ZnS (**a**), Al-ZnS (**b**), Al-ZG0.6 (**c**), Al-ZG1.2 (**d**) and Al-ZG1.8 (**e**).

Figure 6 shows the FTIR transmission spectra of ZnS, Al-ZnS, Al-ZG0.6, Al-ZG1.2, and Al-ZG1.8 composites. The FTIR spectrum of Al-ZG composites present the absorption peaks at 3444 and 1386 cm^−1^, corresponding to O–H stretching vibration of the hydroxyl group. The peaks at 1633 and 1045 cm^−1^ correspond to C=O stretching vibration and C–O symmetric stretching vibration, which are located at the same position of ZnS and Al-ZnS [27]. The appearance of oxygen-containing peaks indicates that the acetate ions were adsorbed on the surface of ZnS, Al-ZnS, and Al-ZG composites [28].

### 3.7. Photocurrent-Time Tests

**Figure 7:**
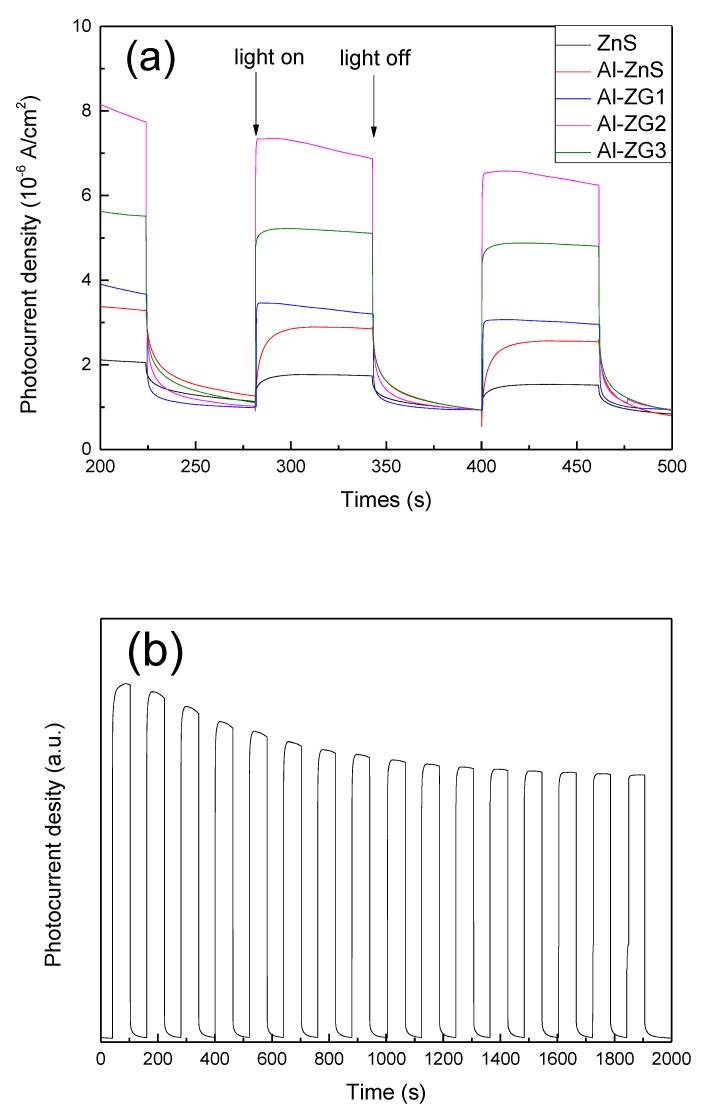
(**a**) Photocurrent-time test of ZnS, Al-ZnS, Al-ZG0.6, Al-ZG1.2 and Al-ZG1.8; (**b**) Photocurrent-time curve of Al-ZG composites in 2000 s.

Figure 7a represents a comparison of photocurrent-time tests of ZnS, Al-ZnS, Al-ZG0.6, Al-ZG1.2, and Al-ZG1.8 composites. Photoelectrons were generated when pure ZnS was illuminated by a light source. A fraction of photoelectrons can be transmitted to form a stable photocurrent, but most of them tend to recombine. To solve the problem, pure ZnS is required to combine with other components to produce a strong stable photocurrent. Compared to pure ZnS, Al-ZnS shows an increase of 145% in photocurrent under the same light illumination due to the decreased energy bandgap and increased photogenerated electrons of Al-ZnS. There are two main reasons for the reduction of the band gap. The first point is due to the exchange interaction between the d electrons of the aluminum ions and the s and p electrons of the host electron band. [11]. The second point is that the addition of aluminum can adjust the chemical state of ZnS and change the mobility and concentration of carrier in the sample, which causes a Burstein–Moss shift and ultimately reduces the band gap [29,30,31]. As shown in Figure 7a, the photocurrent of Al-ZG composites first improves with the increasing GQDs content, reaching a maximum at the GQDs content of 1.2 wt %, and declines with the further increasing of GQD content. This trend is due to GQDs effectively promoting the photoelectrons transfer and preventing the electron-hole recombination. Therefore, a certain proportion of GQDs can increase the photocurrent density of Al-ZG. However, when GQDs content exceeds a certain ratio, the hindrance of light absorption by excessive GQDs leads to a decline in photoelectron generation. The photocurrent density value of Al-ZG is about 700% and 200% of pure ZnS and Al-ZnS, respectively. 

As shown in Figure 7b, the photocurrent density has 20% attenuation and remains stable after thousands. The result suggests that GQDs can separate and store the photoexcited carriers, consequently reducing the recombination rate of the photoexcited electronsand holes, and sustaining the production of photocurrent.

### 3.8. Electrochemical Impedance Spectra

**Figure 8:**
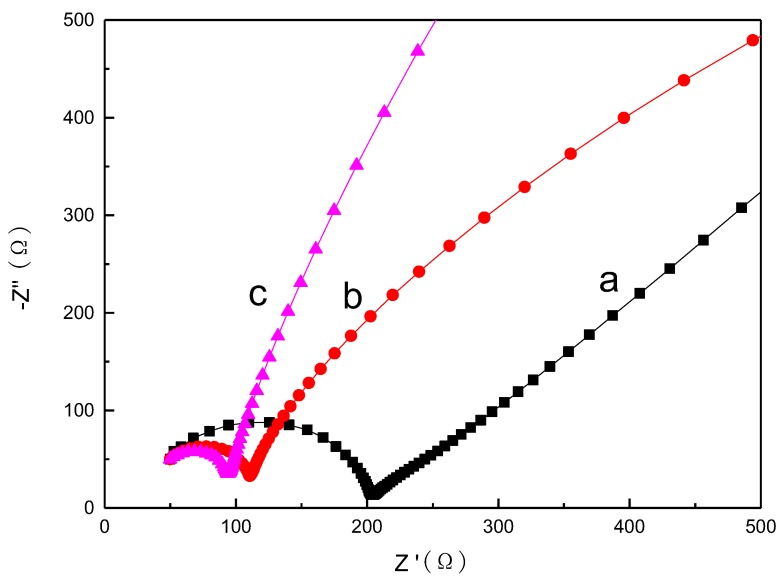
Electrochemical impedance spectra of ZnS (**a**), Al-ZnS (**b**) and Al-ZG1.2 (**c**).

Electrochemical impedance measurements of ZnS, Al-ZnS, and Al-ZG1.2 were investigated in a three-electrode system. A semicircle arc in high frequencies reflects the interface charge-transfer resistance between the electrolyte solution and the working electrode. As shown in Figure 8, the semicircle radius decreased as Al was doped, and further declines as GQDs were incorporated. The result proves that a moderate addition of GQDs can decrease the charge-transfer resistance of Al-ZG.

## 4. Conclusions

A one-step solvothermal process was used to synthesize the Al-ZG composites. The XRD result shows that a small amount of Al doping and GQDs have no obvious change to the cubic ZnS structure. XPS confirms the existence of Al in Al-ZG composites. UV-vis spectra reveal a red-shift from 304 nm to 317 nm with Al doping and present a further red-shift from 317 nm to 332 nm with the incorporation of GQDs. This red-shift might be ascribed to the interaction between GQDs and Al-ZnS [27]. An enhanced luminescence can be observed in Al doped ZnS compared to the pure ZnS. With the addition of GQDs, the luminescence intensity shows a downward trend due to the reason that the GQDs can act as an excellent electron conductor to transfer the electrons to the external circuit and prevent the recombination of electron-hole pairs. The transient photocurrent response of Al-ZnS shows an increase of 145% in photocurrent as compared to pure ZnS due to the decreased energy bandgap and increased photogenerated electrons of Al-ZnS. Al-ZG composites reach a maximum photocurrent at the addition of 1.2 wt %. The photocurrent of Al-ZG is twice larger than that of Al-ZnS due to the lower recombination rate and faster electron-transfer upon the insertion of GQDs.

## Figures and Tables

**Table 1:** Atomic% of elements from the XPS data for the pure ZnS, Al-ZnS and Al-ZG.

Sample	Atomic% (at %)			
	Zn	S	Al	C
ZnS	50.05	49.95	0	0
Al-ZnS	49.37	46.05	2.28	0
Al-ZG	46.28	43.15	2.48	7.55
